# Look Beyond Syncope: A Positive Outcome in the Management of Multiple Bladder Diverticuli-Associated Enterococcus faecium Urinary Tract Infection

**DOI:** 10.7759/cureus.42361

**Published:** 2023-07-24

**Authors:** Ifeoma Kwentoh, Terrence Henry

**Affiliations:** 1 Medicine, Columbia University, New York, USA; 2 Internal Medicine, Harlem Hospital Center, New York, USA

**Keywords:** enterococcus faecium, affordable healthcare, recurrent syncope, elderly falls, congenital bladder diverticula, urinary bladder diverticulum, lower urinary tract symptoms

## Abstract

Enterococcus faecium is a Gram-positive flora bacterium home to the gastrointestinal tracts of humans. A true ubiquitous pathogen and a member of the intestinal microbiome, formerly known as group D streptococci, this pathogen has been around for over 10 centuries. Enterococcus faecium thrives in the presence of stool and sewage. The ability to cause endocarditis and urinary tract infections (UTIs) has led to morbidity and mortality in the adult population. We report a case of an elderly woman who presented with multiple falls to our trauma bay area. She was initially managed as trauma during multiple visits with superficial scalp lacerations. However, with multiple falls, she was subsequently transferred to medicine to rule out cardiogenic versus neurogenic syncope. She was admitted to the telemetry unit, and a cardiologist was consulted. Orthostatic vitals were negative, and she had no fever or leukocytosis. Abdominal computed tomography (CT) done as part of the standard trauma workup revealed an interesting finding of multiple bladder diverticula.

## Introduction

Falls remain listed as the most common presentation in the Emergency Department among the elderly population in the United States [1]. The impact of injury resulting from falls requiring trauma attention can have a significant impact on the quality of life in this baby boomer generation [1,2]. The sheer amount of imaging done as a standard trauma workup and the economic cost to the healthcare system are alarming, raising the question of what modality of screening can be better utilized to improve the approach in the management of frequent falls. Falls can be mechanical or related to systemic illness, such as in cardiac, neurological, and musculoskeletal, or due to an infectious etiology. Infection with certain pathogens such as Enterococcus faecium can result in both urinary tract infection (UTI) and endocarditis [3,4]. Patients often presenting as level 2 trauma in most trauma centers are often transferred to telemetry to rule out syncope as a secondary cause of falls. The incidental findings of other causes of falls may be discovered after several costly and expensive workup. Our article illustrates the need to broaden our differentials of falls in the geriatric population beyond cardiac and neurogenic etiologies. UTI and pneumonia are the top differentials for falls in the elderly. A retrospective study done utilizing a trauma database from a level 1 trauma center in the US concluded that chronic medical conditions are associated with increased mortality and lengthen hospital stay [4]. Early screening for UTI and management will lead to decreased trauma visits in the geriatric population. 

## Case presentation

**Figure 1:**
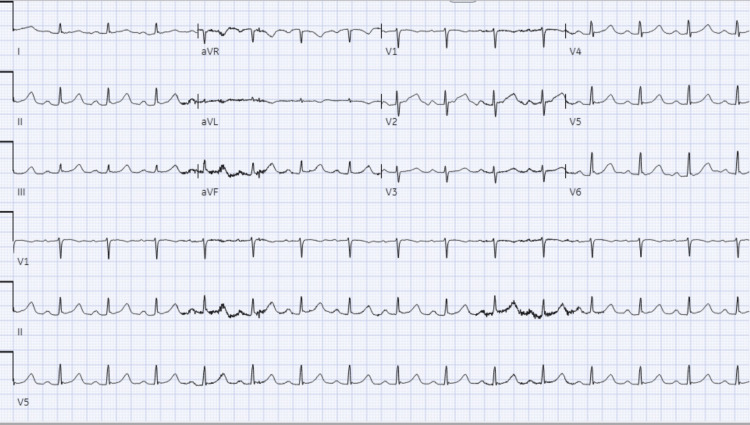
Normal sinus rhythm with prolonged QT.

A 71-year-old female with a past medical history of multiple falls, irritable bowel syndrome, lactose intolerance, pulmonary embolism on Xarelto, sarcoidosis, and Sjogren’s syndrome was admitted as level 2 trauma following another fall. Patient downtime was unknown and unwitnessed. She endorsed falling from a standing position and referred to having blacked out. She denied fever, chills, genitourinary symptoms, cough, or chest pain. There was no history of orthopnea or paroxysmal nocturnal dyspnea. Except for the feeling of lightheadedness and some palpitations, there were no other suggestive symptoms of heart disease or infectious process. On physical exam, she was alert and well-oriented and had minor bruises on her arms and a scalp laceration requiring repair. Orthostatic vitals were negative. The initial primary survey done by the trauma team was completed, and standard trauma imaging for blunt injuries and fractures was ordered. The patient was transferred to telemetry to be worked up for syncope. Labs obtained at initial entry were unremarkable, and urine analysis (UA) showed a trace of leucocyte esterase. However, complete blood count (CBC), liver function test, coagulation panel, and basic metabolic panel were all unremarkable, except for mild elevation in prothrombin time/international normalized ratio in keeping with her anticoagulation. Electrocardiogram (ECG) showed normal sinus rhythm, with premature ventricular contractions (Figure 1).

**Figure 2:**
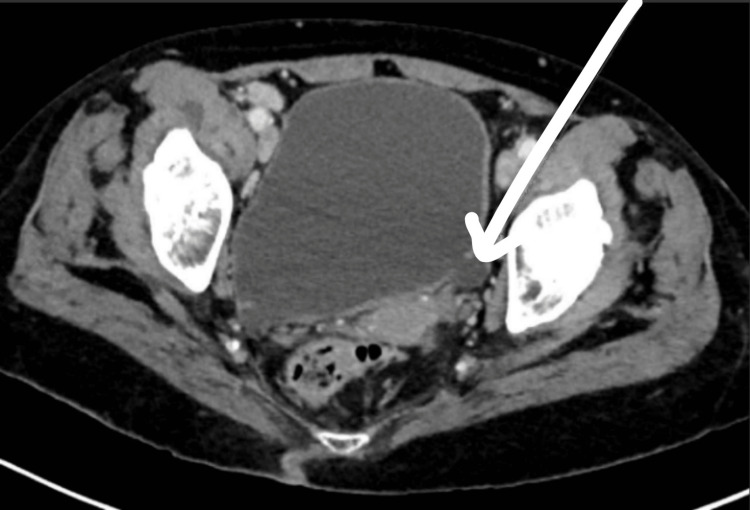
CT abdomen and pelvis with IV contrast showing a left small bladder diverticular; white arrow point inserted to show the mucosa outpouching of the bladder wall. Arrow pointing to the area of the diverticula

**Figure 3:**
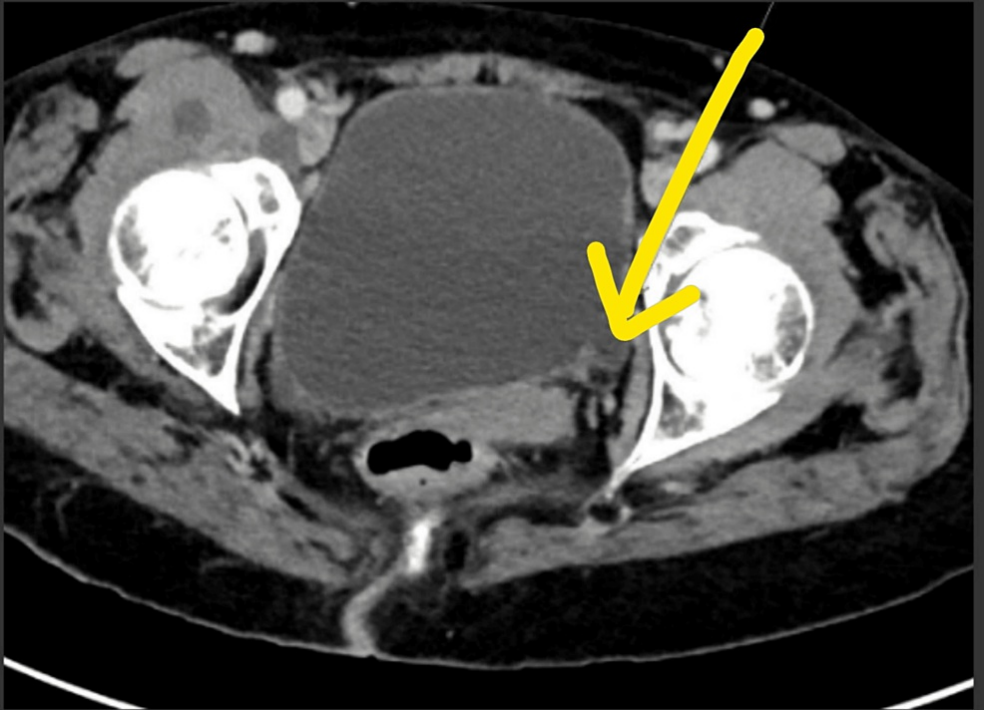
Another left-sided bladder diverticula; yellow arrow inserted to show outpouching of the bladder wall mucosa. CT abdomen and pelvis

**Figure 4:**
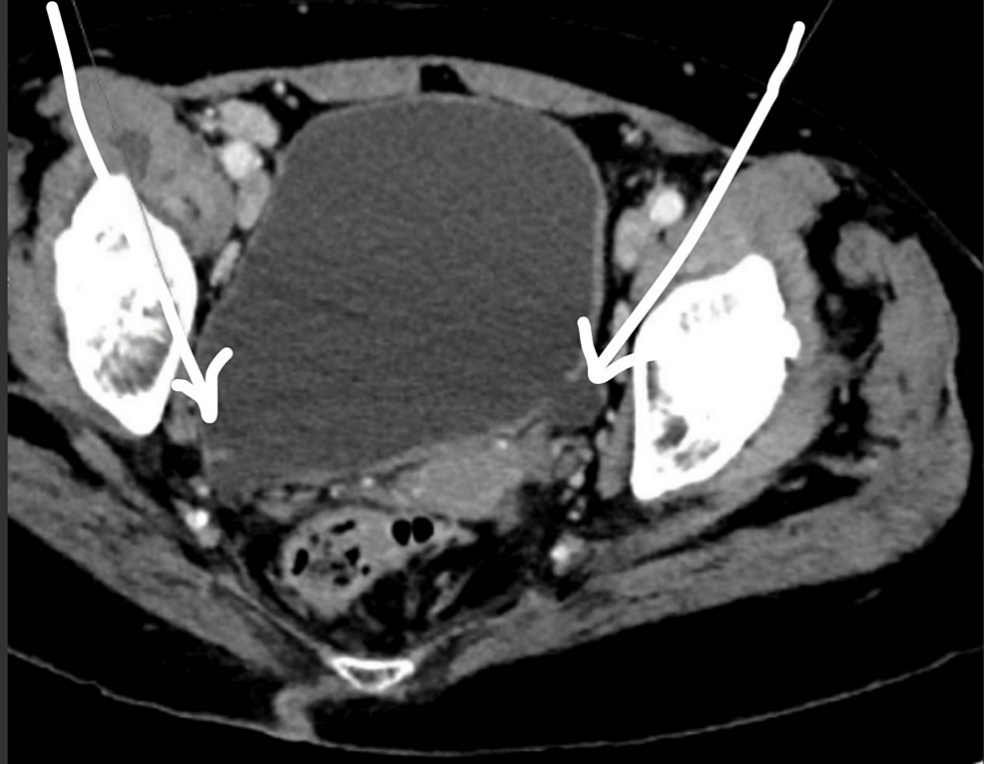
A CT abdomen and pelvis showing left and right multiple bladder diverticula with white arrows inserted demonstrating these multiple diverticula.

Urine culture was pending. Imaging done, including head computed tomography (CT), showed no fractures, except for soft tissue swelling. Spine CT showed degenerative remodeling of the cervical 5 (C5) through cervical 6 (C6) vertebral bodies with slightly decreased vertebral body height and increased anterior-posterior (AP) dimension and no significant central canal stenosis. Chest X-ray was remarkable for magnified cardio mediastinal silhouette, with hilar adenopathy. However, abdominal CT (Figures 2-4) showed multiple bladder diverticula.

Urine culture days following admission revealed Enterococcus faecium sensitive to vancomycin (Table 1).

**Table 1:** Urine microbiology. MIC: Minimum inhibitory concentration, which can be determined by culturing microorganisms in a liquid media or solid growth media. A lower MIC value indicates less drug is required for inhibiting the growth of the organism, and drugs with lower MIC scoring are more effective for the organism cultured.

Susceptibility	Enterococcus faecium (Minimum Inhibitory Concentration, MIC)
Ampicillin	> 8 Resistant
Ciprofloxacin	> 2 Resistant
Levofloxacin	> 4 Resistant
Nitrofurantoin	64 Intermediate
Tetracycline	> 8 Resistant
Vancomycin	1 Sensitive
Final	50,000-99,000 CFU/mL Enterococcus faecium <10,000 CFU/ml normal urogenital flora present

The patient was treated with vancomycin 1.5 g for seven days, and a urologist was consulted, with robotic surgery for trans-vesical diverticulectomy recommended and completed. Discharged and since then has never had another trauma room visit. She had a total of 23 head CTs from 2019 to 2023 following every syncope and fall. A positive outcome was finally achieved that led to the discovery of a relatively uncommon anatomical defect of the bladder and complete recovery. This was a positive outcome in the management of her case.

## Discussion

Bladder diverticula are a rare anatomical outpouching of the mucosa of the bladder wall subepithelial connective tissue and thin muscle fiber [1,2]. They are either congenital or acquired [3,4]. These anomalies are discovered incidentally during a workup for other reasons. Bladder diverticula can cause urinary retention and are often diagnosed on imaging studies, including CT scans or ultrasound [4]. The congenital type mostly occurs in the pediatric population, while the acquired type occurs in adults [5]. These disease entities are mostly prevalent in males than females [6]. Our patient was female with low suspicion of such multiple diverticula. However, after the diagnosis was made and further probing, she referred to the occasional feeling of incomplete emptying with associated lower abdominal fullness and double voiding. Urine culture was positive for a gut flora Enterococcus faecium. She was extensively worked up for cardiogenic, neurogenic, and Infectious etiologies for multiple falls. The approach to managing the diverticula of the bladder is expectant treatment, except when it causes multiple UTIs, malignancy, or stones, then laparoscopic robotic surgeries for transcervical removal of the diverticula are usually recommended. This case broadened our differentials to syncope, acknowledging that not all falls are syncope in the elderly. The economic burden of this pathogen in the geriatric population contributes to expensive healthcare costs [7,8]. Patients also reported occasional diarrhea, during the course of these falls; academic discussions have focused on the mechanism of translocation of this normal flora of the gastrointestinal tract to the bladder. This area requires further research. The finding of the multiple bladder diverticula solved the mystery. One may argue that falls can occur as a result of multifactorial reasons, Frequent UTIs occur in the geriatric population, with Escherichia coli being the most cultured organism [8]. This case is unique as most management of UTIs is centered around antibiotics; this was an anatomical defect that required intervention and overall reduced trauma bay visits and the cost to the healthcare system.

## Conclusions

A positive outcome in the management of syncope should include thorough differentials inclusive of infectious etiology. Our patient was recycled for many months in the healthcare system until a definitive diagnosis of this rare multiple bladder diverticula was found and managed appropriately. A total of 23 head CT scans were completed throughout her multiple visits to different hospitals, including our emergency room and trauma bay, during that time. Bladder diverticula are, majority of the time, an incidental finding on imaging investigations done for other reasons. They can be found in different imaging types, including ultrasound, CT, and MRI. Physicians should have a high index suspicion for infectious causes of syncope.
